# Bilateral rapidly destructive hip disease: a case report and literature review

**DOI:** 10.3389/fmed.2025.1630171

**Published:** 2025-08-14

**Authors:** Yan Shen, Yi Zhang, Jing Fu, Hang Pei, Jiang Hua, Bangjian He

**Affiliations:** ^1^Huzhou Zhengbo Orthopedic Hospital, Huzhou, Zhejiang, China; ^2^Zhejiang Chinese Medical University, Hangzhou, Zhejiang, China; ^3^Department of Stomatology, Xixi Hospital, Hangzhou, Zhejiang, China; ^4^Department of Orthopedics, The First Affiliated Hospital of Zhejiang Chinese Medical University, Hangzhou, Zhejiang, China

**Keywords:** bilateral rapidly destructive hip disease, total hip arthroplasty, rheumatism, case report, literature review

## Abstract

Rapidly destructive hip disease (RDHD) is a rare syndrome marked by swift joint degeneration and substantial functional impairment, typically affecting a single hip joint. The occurrence of bilateral RDHD is exceptionally uncommon, highlighting the urgent need for further research into its pathogenesis, pathological changes, and treatment strategies. We present the case of a 75-year-old female who developed significant bilateral hip mobility limitations and buttock pain 6 months after being diagnosed with mild bilateral hip osteoarthritis. Imaging revealed the disappearance of both femoral heads. After excluding contraindications, the patient successfully underwent left uncemented total hip arthroplasty (THA). Postoperative pathology confirmed the diagnosis, showing degeneration and focal necrosis. This case represents the first documented instance of favorable clinical outcomes following THA in bilateral RDHD.

## Background

Rapidly destructive hip disease (RDHD) is a rare syndrome characterized by rapid joint degeneration without a specific diagnosis. It differs significantly from typical arthritis and osteonecrosis, presenting a short course, swift femoral head destruction, and profound functional loss, which is infrequent in clinical practice. RDHD was first reported by Forestier in 1957 and later standardized by Lequesne in 1970; however, its etiology and pathogenesis remain elusive. RDHD primarily affects elderly women, with unilateral hip joint involvement observed in about 80%–90% of cases (1). According to existing literature, the pathological changes associated with RDHD typically manifest over a duration of 6 weeks to 16 months from the initial appearance of symptoms (2–4). Our research objective is to report a clinical case of bilateral rapidly destructive hip disease and provide a comprehensive summary of the unique characteristics of this condition based on an extensive review of relevant literature.

## Case presentation

**FIGURE 1:**
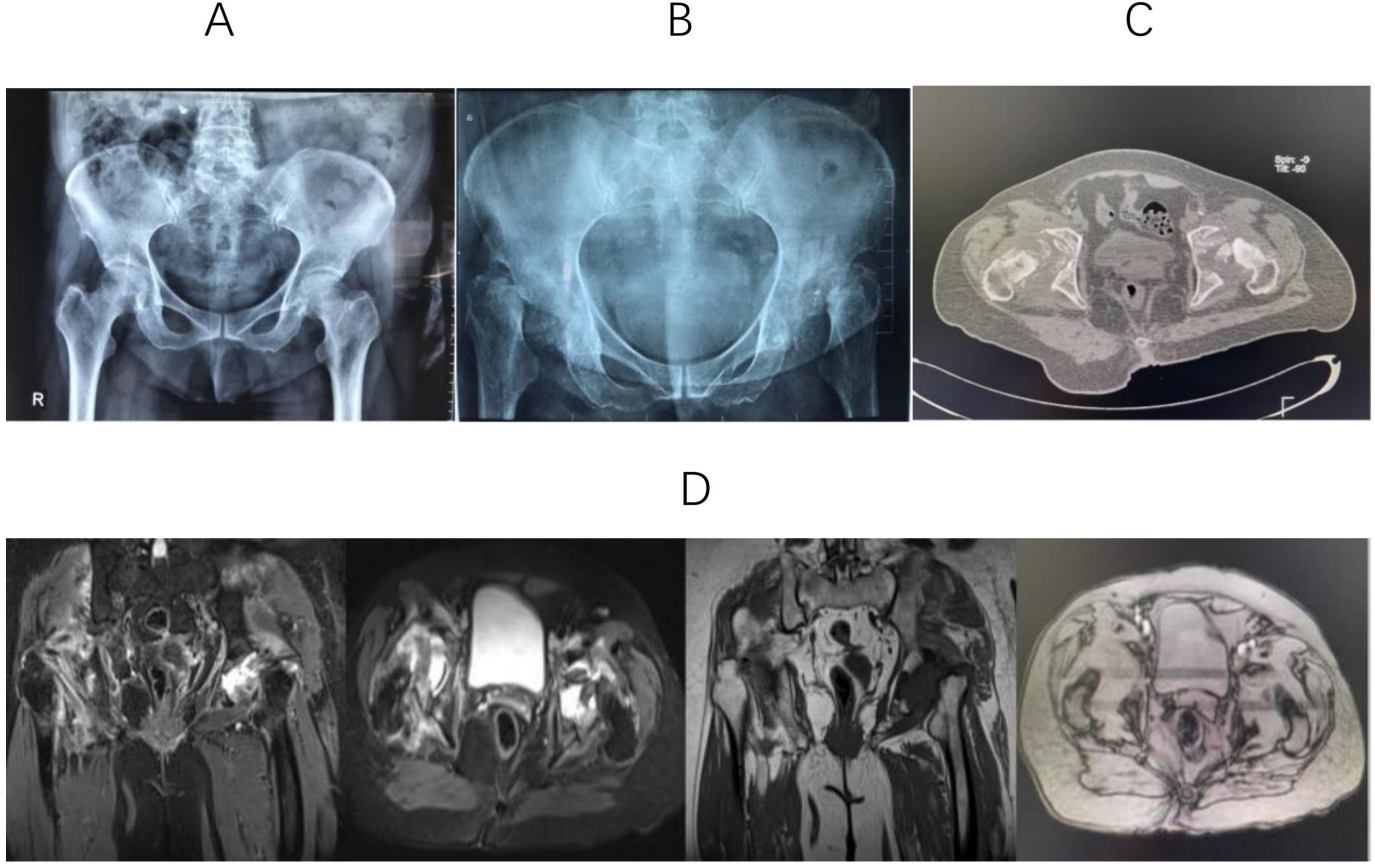
**(A)** X-ray shows that the joint space of both hips decreases, and the density of the bone cortex increases. **(B)** Anteroposterior hip radiographs revealed near-total femoral head destruction, uneven acetabular bone density, and bilateral hip dislocation. **(C)** CT shows that high-density patchy shadows are scattered in the femoral head area, and the acetabulum on both sides is shallow. **(D)** MRI showed bilateral destruction of the femoral heads and acetabulum, with thinned acetabular cartilage and loss of femoral head shape. Abnormal signal shadows appeared as hypointense on T1-weighted sequences and hyperintense on short TI inversion recovery scans.

A 75-year-old female patient presented 6 months ago with bilateral hip pain, and pelvic X-rays (Figure 1A) confirmed mild bilateral hip osteoarthritis. Two months ago, the patient experienced worsening bilateral hip pain with progressive mobility impairment, without any history of trauma or steroid use. The patient had a medical history of hypertension, atrial fibrillation and type 2 diabetes mellitus. She had no known history of chronic kidney disease, endocrine disorders, or other metabolic bone diseases prior to admission, and denied any history of corticosteroid use. Imaging (Figures 1B–D) revealed the disappearance of bilateral femoral head structures. Laboratory tests ruled out infectious, malignant, and inflammatory diseases. Based on bone density and metabolic assessments, a diagnosis of bilateral hip RDHD and osteoporosis was confirmed. The patient’s baseline characteristics and relevant examination data are presented in Table 1.

**TABLE 1:** Basic information of the patient and relevant examination data.

Project	Data	Normal range	Unit
BMI	19.3	18.5–24	kg/m^2^
Bacterial culture of joint effusion	–	–	/
Bone mineral density (*T* score)			
Left hip	−2.8	>−2.0	/
Lumbar spine	−2.9		
Bone metabolism index			
Osteocalcin	19.24	8.87–29.05	ng/ml
25(OH)D	14.91	30–100	ng/ml
PINP	152.50	21.32–112.80	ng/ml
β-CTx	1247.80	131–900	pg/ml
D-dimer	0.27	0.00–0.55	mg/L

25(OH)D, 25-hydroxyvitamin D; PINP, procollagen type I *N*-terminal propeptide; β-CTx, beta-isomer of C-terminal telopeptide of type I collagen.

**FIGURE 2:**
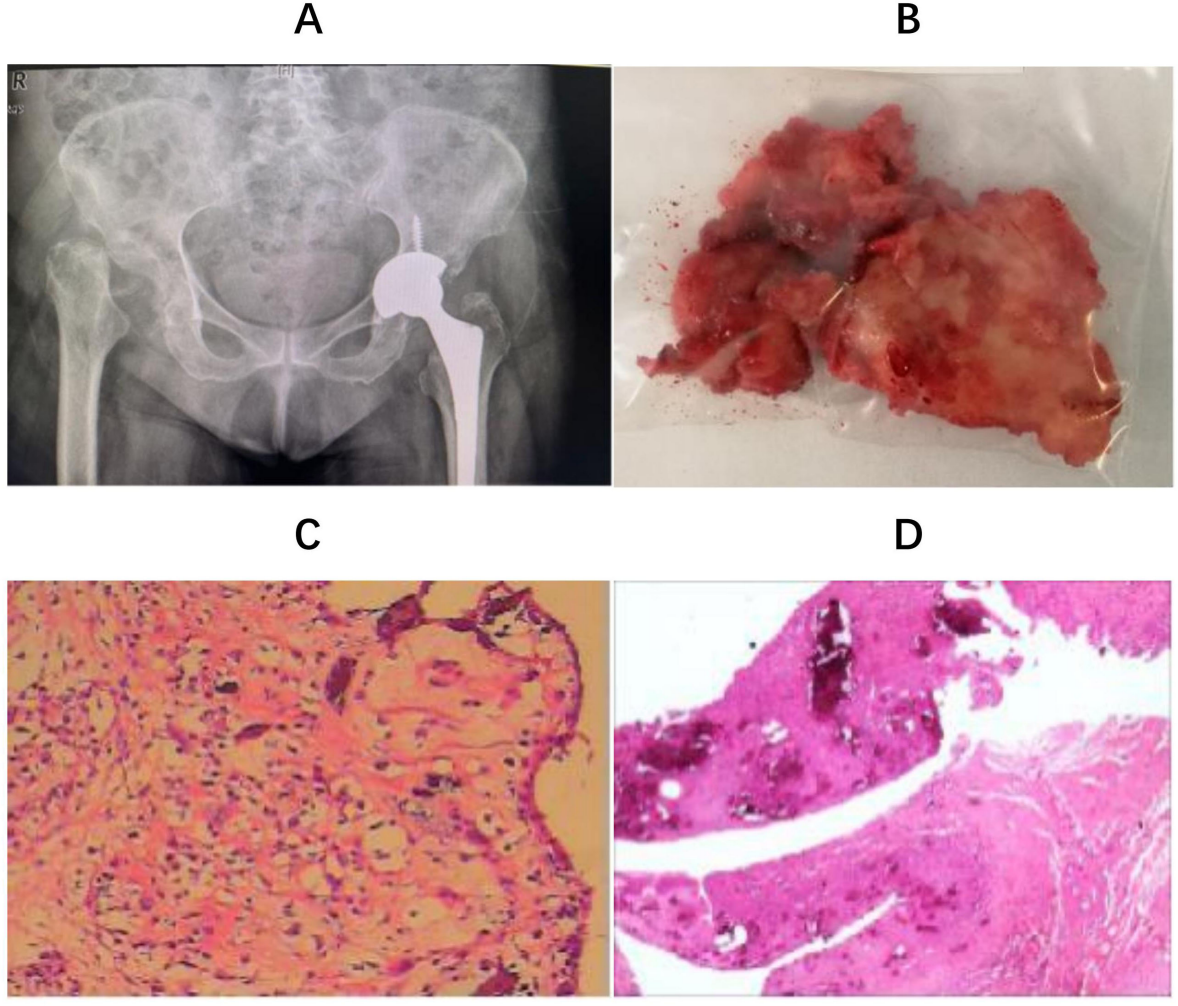
**(A)** The prosthesis used a Zimmer, Trilogy IT cup and FMT proximal porous-coated tapered titanium stem with ceramic to polyethylene surface bearings (Zimmer, Blolox Delta ceramic femoral head); **(B)** remaining femoral head tissue; **(C)**: intraoperative frozen biopsy suggested synovial hyperplasia of the “left hip joint” with chronic inflammatory cell infiltration; **(D)** histological examination reveals pathological diagnosis of RDHD.

Considering the patient’s advanced age and multiple underlying medical conditions, a staged total hip arthroplasty was selected after discussion with the patient and her family, with a 3-month interval between the procedures. After excluding contraindications, the patient underwent a left uncemented total hip arthroplasty (THA) (Figure 2A). Intraoperatively, a large amount of turbid fluid and residual cartilage fragments from the femoral head were observed within the joint capsule (Figure 2B), with the femoral head entirely destroyed. Additionally, there was pronounced edema of the periarticular tissues, accompanied by significant synovial proliferation and edema (Figure 2C). Postoperative pathological examination of the residual tissue revealed synovial and fibrous hyperplasia, with evidence of inflammatory cell and histiocyte infiltration, granuloma formation, and degeneration of fibrous and cartilaginous tissue. Additionally, calcification, ossification, and areas of bone degeneration with focal necrosis were observed (Figure 2D), consistent with the pathological diagnosis of RDHD.

Following surgery, the patient’s left hip pain was resolved. She stood by the second postoperative day and initiated assisted ambulation with a walker by day four. Three months postoperatively, she was hospitalized in the respiratory department due to a COVID-19 infection and subsequently experienced an acute myocardial infarction. After evaluation by the anesthesiology team, the surgical risk was deemed high. Considering this, the patient and her family opted for conservative management of the contralateral hip. Despite these complications, the patient was able to ambulate with the assistance of a walker during the postoperative period. A chronological summary of the patient’s clinical course is presented in Table 2.

**TABLE 2:** A chronological summary of the patient’s clinical course.

Time point	Event
6 months before admission	Onset of bilateral hip pain
2 months before admission	Worsening symptoms
Admission	Combined with imaging examinations, RDHD was diagnosed.
Day 0	Left THA performed
Postoperative day 2	Stood with assistance
Postoperative day 4	Began assisted ambulation with walker
3 months postoperative	COVID-19 infection and hospitalization
During hospitalization	Acute myocardial infarction occurred
Final follow-up	Conservative treatment

RDHD, rapidly destructive hip disease; THA, total hip arthroplasty.

## Discussion and conclusions

The formalized characterization of RDHD was introduced by Lequesne in 1970, delineating it as chondrolysis exceeding 2 mm within a year or a 50% reduction in joint space within a year, while concurrently lacking indications of alternative forms of expeditiously deteriorating arthropathy. RDHD frequently encounters misidentification as alternative conditions including hip osteoarthritis, femoral head osteonecrosis, seronegative arthritis, neuroarthropathy, rheumatoid arthritis, or tumor-related ailments (5). The majority of RDHD occurs in elderly women affecting a unilaterally hip; bilateral involvement is rare. Lintao Hu’s (6) systemic literature review collected reported studies (181 cases), and only three cases (1.7%) were bilateral arthropathy. The prognosis of RDHD largely depends on the timing of diagnosis, the extent of joint destruction, and the patient’s general condition. Okamoto et al. (7) found that RDHD patients reported lower satisfaction after THA than those with OA; however, timely THA still yielded significant pain relief and functional improvement. In this case, a 75-year-old woman developed bilateral RDHD but was initially misdiagnosed with common hip osteoarthritis, leading to rapid joint destruction. Although only unilateral THA was performed due to medical concerns, the patient regained partial mobility. This highlights the importance of early recognition and surgical intervention in RDHD.

Although the exact cause remains elusive, several potential risk factors have been linked to RDHD. Various studies (8–10) have suggested the involvement of certain medications, including indomethacin toxicity, prolonged corticosteroid usage, and certain anticancer agents. Takamori (10) documented an exceptional instance of RDHD potentially linked to crizotinib, occurring in a patient diagnosed with ROS1-positive lung adenocarcinoma. Intra-articular steroid injection is another potential risk factor. Tiwari A (11) documented a case of RDHD in a 62-year-old woman who developed the condition within 2 months of receiving an intra-articular steroid injection. Hess (12) highlighted a 21% occurrence of RDHD of the hip in individuals undergoing intra-articular steroid injections, emphasizing that careful evaluation of the cost-effectiveness and safety of this treatment is essential, especially for elderly patients or those with higher preinjection KL scores. Subchondral insufficiency fracture of the femoral head has been proposed as a potential underlying cause of RDHD (13).

Although histological evidence of subchondral insufficiency fracture might be present, the precise mechanism remains uncertain. Fukui (14) proposed that the inversion of the labrum is a prerequisite for subchondral insufficiency fracture and demonstrated these observations in the early stages of the disease as a potential mechanism contributing to RDHD. Sagittal spinopelvic alignment may influence the development of hip diseases. Morimoto (15) examined the sagittal spino-pelvic alignment in female patients with RDHD and osteoarthritis. They observed that patients with RDHD exhibited a notable increase in posterior pelvic tilt along with reduced lumbar lordosis, lumbar range of motion, and sacral slope when compared to those with osteoarthritis. This implies that sagittal spino-pelvic alignment could potentially contribute significantly to the onset of RDHD.

Previous studies have investigated the molecular basis of RDHD. Komiya et al. (16) reported elevated levels of prostaglandins, interleukin-1β, and matrix metalloproteinases (MMP-2 and MMP-3) in the synovial fluid of RDHD patients compared to those with typical osteoarthritis, suggesting an aggressive inflammatory and degradative process. Yasuda et al. (17) further refined RDHD classification based on imaging findings, identifying a subtype (type 2) characterized by rapid femoral head destruction within one year, which was associated with increased serum MMP-3 levels and posterior pelvic tilt. In subsequent work, higher levels of bone turnover markers–tartrate-resistant acid phosphatase 5b and bone alkaline phosphatase–were also linked to this aggressive subtype, indicating that these markers might help predict early structural damage (18). In our case, the patient exhibited markedly elevated levels of bone turnover markers, including PINP (152.50 ng/ml) and β-CTx (1247.80 pg/ml), suggesting active bone metabolism. These findings support the potential utility of serum markers in identifying patients at risk for rapid joint destruction, although further validation is needed before clinical application.

Imaging remains the primary diagnostic tool for RDHD. Blood tests like CRP and ESR lack specificity in this context. In reported cases, X-rays served as the initial radiological assessment, enabling physicians to accurately diagnose RDHD (6). Initial radiographs for RDHD typically involve hip or pelvic plain radiography. These often exhibit no evident positive findings, occasionally indicating mild hip degenerative arthritis. Any osteophytes present tend to be small, and subchondral sclerosis is minimal. In our case, the initial pelvic radiographs revealed slight osteoarthritis in both hips (Figure 1A). Late-stage RDHD is primarily characterized by extensive destruction of hip joint structures on X-rays. The characteristic advancement, akin to our case, involves near-total femoral head disintegration, asymmetrical elevation in acetabular bone density bilaterally, and dislocation of both hips (Figure 1B). In certain patients, pelvic X-rays reveal remnants of the femoral neck after the complete absorption of the femoral neck (19). Available studies on early-stage RDHD are lacking in CT data. Research by Yasuda (17) demonstrated mild arthritis or partial femoral head destruction on CT. During the middle and advanced stages, CT findings mirror those of plain radiographs. The femoral head and neck may be entirely absorbed, leaving the acetabulum filled with low-density tissue, and noticeable soft tissue swelling around the joint. The CT findings of our case (Figure 1C) were consistent with previous studies (19, 20). Elaborating on early-stage RDHD MRI findings, Boutry (21) and Sugano (22) outlined prominent characteristics: joint effusion and femoral head, neck, or acetabulum displaying edema-like patterns. Furthermore, Fukui (4) noted a distinctive low-intensity band in T1-weighted images. Watanabe (13), investigating MRI results of RDHD patients a month after hip pain onset, identified subchondral bone edema in the femoral head’s stress region. Late-stage RDHD MRI images reveal modest joint fluid accumulation, fragmented femoral head articular cartilage, and irregular signals within the femoral head. As the disease progresses, there may be an increase in fluid accumulation within the hip joint (22). Late-stage RDHD is characterized by femoral head and neck destruction, uneven remnants of the femoral head and neck, significant thickening of the hip capsule, and presence of free bone tissue within the joint capsule. MRI findings obtained 6 months after the initial office visit in this case were in line with previous reports. Furthermore, we identified bilateral acetabular destruction (Figure 1D).

Several conditions could be manifested as the presentation of rapid destructive hip disease, including infection, malignancy, sarcoidosis, Charcot or neuropathic arthropathy, inflammatory arthritis, osteonecrosis, hemophilia, and ochronosis (23, 24). In cases where joint destruction progresses swiftly, as in our situation, it becomes imperative to exclude other potential diseases and to entertain the possibility of a diagnosis of RDHD. There are several scenarios that warrant consideration of RDHD: (1) Female gender aged over 70 years, experiencing severe hip pain for less than a year; (2) Hip pain that does not significantly alleviate or worsens after receiving intra-articular steroid injections; (3) Rapid and substantial femoral head destruction or joint space narrowing at a rate exceeding 2 mm/year; (4) Exhaustive exclusion of other potential causes for severe hip joint destruction.

Late-stage RDHD is typically managed with total hip arthroplasty (25). Hart (24) has suggested a joint replacement strategy for RDHD to mitigate the potential drawbacks of an unnecessary two-stage procedure. In cases of rapidly progressing atrophic bone loss evident on radiographs, alongside elevated ESR and CRP levels, consideration should be given to performing a hip aspirate. If the results of these assessments do not align with an infection, THA could be pursued, irrespective of the appearance of intra-articular fluid. In cases where an aspirate was not obtained and concerns persist regarding the appearance of joint fluid during THA, intraoperative measures such as cell count, frozen section analysis, or leukocyte esterase dipstick testing can provide valuable guidance for management decisions. This strategy can help resolve the uncertainty of proceeding with arthroplasty when encountering rapidly progressive joint destruction. Preoperative blood tests and synovial fluid cultures conducted on our patient ruled out joint infection. Subsequently, she underwent left THA. During the procedure, significant turbid fluid was noted within the joint capsule, along with an abundance of necrotic bone and fragmented cartilage tissue. Notably, the femoral head exhibited substantial dissolution, leaving behind only remnants of cartilage fragments (Figure 2B). Additionally, pronounced edema was observed in the surrounding tissues, often accompanied by prominent synovial hyperplasia and edema. Intraoperative frozen biopsy (Figure 2C) of the affected area indicated synovial hyperplasia in the left hip joint, with infiltration of chronic inflammatory cells. Subsequent histological analysis confirmed the diagnosis of RDHD (Figure 2D). Following the surgical intervention, the patient experienced alleviation of pain in her left hip joint. However, while THA is the established treatment approach, it is essential to acknowledge the potential for the disease progression to persist, which could potentially jeopardize the integrity of the prosthesis.

A comprehensive literature review was undertaken (25), revealing midterm outcomes (average: 5 years) after THA. The findings indicated favorable-to-excellent clinical results (clinical hip scores > 80 points) and a revision rate of 3%. These results underscore the significant efficacy of THA as a valuable therapeutic avenue for individuals afflicted by rapidly advancing hip conditions. The underlying pathological progression does not seem to compromise the longevity of the implant. Remarkably, blood loss during total hip arthroplasty in RDHD surpasses that observed in routine cases of osteoarthritis and conventional osteonecrosis of the femoral head (ONFH) (26). The survival rate of THA at the 5-year mark exceeds 95%, regardless of whether cemented or non-cemented prostheses are employed in cases of RDHD (27, 28). Given this, we advocate for the adoption of THA upon definitive diagnosis confirmation, as this approach not only mitigates profound joint degradation but also simplifies the surgical procedure and reduces the risk of substantial blood loss.

## Data Availability

The original contributions presented in this study are included in this article/supplementary material, further inquiries can be directed to the corresponding author.
